# Farmer Behavior and Gastrointestinal Nematodes in Ruminant Livestock—Uptake of Sustainable Control Approaches

**DOI:** 10.3389/fvets.2018.00255

**Published:** 2018-10-16

**Authors:** Fiona Vande Velde, Johannes Charlier, Edwin Claerebout

**Affiliations:** ^1^Laboratory of Parasitology, Department of Virology, Parasitology and Immunology, Faculty of Veterinary Medicine, Ghent University, Merelbeke, Belgium; ^2^Department of Communication Studies, Faculty of Political and Social Sciences, Ghent University, Gent, Belgium; ^3^Kreavet, Kruibeke, Belgium

**Keywords:** gastrointestinal nematodes, sustainable nematode control, dairy farmers' behavior, social veterinary epidemiology, targeted communication

## Abstract

Gastrointestinal nematode (GIN) infections are a common constraint in pasture-based herds and cause a decrease in animal health, productivity and farm profitability. Current control practices to prevent production losses of GIN infections in livestock depend largely on the use of anthelmintic drugs. However, due to the continued use of these drugs over more than three decades, the industry is now increasingly confronted with nematode populations resistant to the available anthelmintics. This emerging anthelmintic resistance (AR) in cattle nematodes emphasizes the need for a change toward more sustainable control approaches that limit, prevent or reverse the development of AR. The uptake of diagnostic methods for sustainable control could enable more informed treatment decisions and reduce excessive anthelmintic use. Different diagnostic and targeted or targeted selective anthelmintic control approaches that slow down the selection pressure for anthelmintic resistance have been developed and evaluated recently. Now it is time to transform these insights into guidelines for sustainable control and communicate them across the farmer community. This article reviews the current uptake of such sustainable practices with a focus on farmer's socio-psychological factors affecting this uptake. We investigate communication as a possible tool to change current behavior and successfully implement more sustainable anthelmintic treatment strategies.

## Introduction

Gastrointestinal nematode (GIN) infections are a common constraint in pasture-based herds and can cause a decrease in animal health, productivity and farm profitability. Current control practices to prevent production losses of GIN infections in livestock depend largely on the use of anthelmintic drugs. However, due to the continued use of these drugs, the industry is increasingly confronted with anthelmintic drug-resistant nematode populations. This emphasizes the need for sustainable treatment approaches that minimize the selection pressure and spread of anthelmintic resistance (AR). The uptake of methods for sustainable worm control could enable more informed treatments and reduce excessive anthelmintic use. Accordingly, in order to successfully implement such control strategies and change the behavior of farmers, their current perceptions and behaviors need to be comprehended and translated into effective communication strategies.

This review presents a brief history of GIN control practices in developed countries and how the field should shift toward more sustainable control approaches. It gives a critical overview of the behavioral literature in the field, which contributed to understand the limited uptake of these sustainable practices, followed by possibilities for improving this currently ill-equipped domain of behavioral research. Finally, additional research on communication practices is suggested, such as knowledge exchange, since this may bridge the gap between scientific knowledge and applicable advices.

## Current status of gastrointestinal parasitism

### The effects of gastrointestinal parasitism in cattle

All grazing livestock are exposed to GIN infections, which can cause parasitic gastroenteritis. This disease typically affects young animals during their first grazing season and provokes clinical signs, such as diarrhea, reduced growth and weight loss. In severe cases it can cause mortality. Due to their immunity, adult cows generally present no clinical signs, but diminished milk and meat production can be attributed to sub-clinical infections. Consequently, substantial economic losses are due to GIN infections in dairy farms in developed countries (1–4). Today, some authors estimate GIN infections to be second to mastitis in terms of health costs to dairy farms (5).

### Anthelmintic use and farm intensification: co-evolution

The development and availability of highly efficacious anthelmintic drugs has significantly contributed to reducing the economic burden of GIN infections (6, 7). There are three major anthelmintic classes licensed in northern Europe for the control of parasitic nematodes in cattle: benzimidazoles (e.g., fenbendazole), imidazothiazoles (e.g., levamisole,), and macrocyclic lactones (e.g., ivermectin), and these are all used preventively for GIN control. This practice can be put in the “*zeitgeist*” of the late 1980s, where animal health management shifted from treatment of clinical illness of a single animal to disease prevention on a herd level (8). To understand this change of practice, we have to take a better look at the industry. Farming became an agricultural production business included in the global economy. Farm intensification led to drastic changes regarding animal disease control. In the case of GIN control, anthelmintics have been used extensively to prevent emerging infections and thus economic losses. Anthelmintic drug development and the strategic use positively balanced the economic equation (9, 10). This arsenal of relatively inexpensive and highly effective drugs was used to maximize livestock health, productivity and profitability but also led to parasite control that was almost merely based on the frequent use of anthelmintics (11). Their ease-of-use was an excellent and often cheaper substitute for other, more labor-intensive control approaches based on extensive grazing (less animals per hectare) or rotation management for example. Hence, the changing industry and the effectiveness of anthelmintic drugs resulted in an approach that was highly successful, but now new drivers urge for adaptations to the current practices. Moreover, the farm intensification model is also facing a paradigm shift. Due to growing needs for sustainable intensification; i.e., a process or system where agricultural yields are increased without adverse environmental impact and without the conversion of additional non-agricultural land, (12), and changing market demands (e.g., organic, local produce), disease control approaches based on intensive drug use are being pressured toward new practices that include environmental and animal welfare objectives (13), and result in decreased frequency of anthelmintic treatment with a focus on curative practices.

### Anthelmintic resistance in ruminants

Today, the industry is increasingly threatened by populations of nematodes resistant to the most commonly used anthelmintic drugs (14). Resistance to almost every marketed anthelmintic against nematodes in ruminants has developed worldwide, see Table 1 for an overview (15). The rapid acceptance and widespread use of anthelmintics led to an increased series of reports on AR in small ruminants in the 1990s. This had elevated the issue of AR from being a potential problem of the future to being a major threat to small ruminant production in many countries (11, 16). In the cattle industry AR appears to have developed more slowly than in small ruminants (17). However, the increasing number of reports over the past years suggests a rapidly escalating problem (14), with growing numbers of failures of anthelmintic drugs to control cattle nematode parasites all over the world (18–22). Moreover, concern rises when we consider the fact that levels of resistance can increase abruptly (23).

**Table 1:** Introduction of anthelmintic drugs and development of resistance.

**Anthelmintic class**	**Generic name**	**Market release**	**Reported resistance**
Heterocyclic compounds	Phenothiazine	1940	1957
	Piperazine	1954	1966
Benzimidazoles	Thiabendazole	1961	1964
	Cambendazole	1970	1975
	Oxibendazole	1970	1985
	Mebendazole	1972	1975
	Albendazole	1972	1983
	Fenbendazole	1975	1982
	Oxfendazole	1976	1981
	Triclabendazole	1983	1998
Imidazothiazoles and	Levamisole	1970	1979
Tetrahydropyrimides	Pyrantel	1974	1996
	Oxantel	1976	/
	Morantel	1970	1979
Macrocyclic lactones	Abamectin	Late '70	2001
	Ivermectin	1981	1988
	Moxidectin	1991	1995
	Doramectin	1993	2007
	Eprinomectin	1996	2003
Amino-acetonitrile derivate	Monepantel	2009	2013

*Table adapted from De Graef et al. (15)*.

New anthelmintics will be developed in the future. However, recent and probably continuing consolidation of the animal health industry means that fewer and fewer resources are devoted to anthelmintic R&D (24). Likewise, the cost of developing new anthelmintics can be an important barrier to the development of new drugs. Moreover, new actives (i.e., monepantel and derquantel) have been put in the market for sheep recently, but the first cases of resistance are now being reported from various regions (25, 26). Therefore, it is unrealistic to presume that sufficient numbers of new drugs will be developed to maintain a control paradigm based solely on frequent anthelmintic treatment (11), and the need for GIN control practices that slow the development of AR is rising rapidly (27–29).

## Best practice management

### Novel approaches for GIN control in ruminants

The advent of AR is driving the development of new control practices in livestock farming. Such new practices are set up to preserve the efficacy of current and any possible future drugs. These novel approaches should rather sooner than later replace or complement control practices that rely solely on uninformed and repeated treatment of animals.

Two important approaches have been proposed to use anthelmintics in ruminants in a sustainable way. A first approach is the use of combinations of different anthelmintic classes with nematocidal activity (30, 31). Although this strategy can delay the onset of AR, a recent experience suggests that it is unsustainable if the way in which the drugs are used remains unchanged (32); e.g., resistance to the new combination products could develop at the same time (33, 34). The second approach is based on “*refugia*” strategies, which are based on the concept that the rate of AR development is slowed by maintaining a proportion of the parasite population unexposed to anthelmintic drugs (35). *Refugia* is the proportion of the worm population that is not selected by drug treatment and the bigger this proportion, the slower AR will develop (36). The challenge that exists is in finding the best proportion of *refugia* to minimize the AR development, whilst maintaining acceptable animal performance. Two methods are considered to optimize treatment (28); targeted treatments (TT; the whole group of animals is treated after diagnostic information) and targeted selective treatment (TST; treatments directed only to individual animals within a group based on diagnostic information on the individual animal level). This relatively novel approach depends primarily on the use of different parasitological, pathophysiological and/or immunological markers (see Table 2 for an overview), and only secondly on the implementation of anthelmintics to the targeted (group of) animals. The implementation of sustainable practices, such as TT and TST has been proven effective throughout empirical scientific studies and in commercial settings (37).

**Table 2:** Evidence-based indicators to support targeted (TT) and targeted selective (TST) anthelmintic treatments against gastrointestinal nematodes in ruminants.

	**Young cattle**	**Adult cattle**
TT indicators	Grazing management	Grazing management
	Mean FEC after 4–8 weeks during first grazing season	Bulk tank milk anti *Ostertagia ostertagi* antibody level
	Mean serum pepsinogen level at end of grazing season and/or housing	Time of effective contact with gastrointestinal nematode larvae based on qualitative analysis of grazing history until first parturition
TST indicators	Live weight gain	
	Body condition score in combination with FEC	

*Table adapted from Charlier et al. (27)*.

Other alternative methods to control GIN infections that do not require the use of anthelmintic drugs include vaccination, genetic selection, biological methods and pasture management (38). Pasture management, since long advocated as alternative approach to interrupt the life cycle of the nematodes and reduced exposure to infection, is the most feasible non-chemotherapeutic control measure at this moment (1, 39–41). However, grazing management strategies, such as pasture resting, mowing, late turn-out, stock rotation, etc. demand much effort and are sometimes limited due to availability of grassland or other resources. Moreover, these practices require good epidemiological knowledge, which is not always available to the farmer. Therefore, an integrative approach of grazing management and implementation of anthelmintics by targeted decision making is to date recommended as the most feasible and sustainable GIN control method (32).

### Limited uptake of new strategies

Due to increasing reports of AR, guidelines and extension programs were created to promote sustainable worm control, such as Sustainable Control of Parasites in Sheep (SCOPS) for small ruminants and Control of Worms Sustainably (COWS) for cattle in the UK (42), and Wormkill, WormBoss (43) for small ruminants in Australia, to name a few. These initiatives are a collaboration between interested parties from across all sectors of the industry with a view to developing guidelines intended to develop and promote practical recommendations for producers and advisors (44). The recommendations are based on a range of different approaches, and promote “best practice” control for the preservation of current and future anthelmintics. For cattle, COWS recommendations are summarized into eight guidelines presented in Table 3. Learmount et al. (45, 46) evaluated a 3-years implementation of the SCOPS guidelines on commercial sheep farms in the UK. They found a significant reduction in anthelmintic treatments without loss of animal performance, confirming the effectiveness of such advices in the field (45). Similarly, in Brazil, the assessment of the FAMACHA^©^ system (method for assessing ocular membrane coloration as an indicator of hemonchosis in small ruminants) resulted in a decrease of anthelmintic administration (47–49). Nevertheless, the uptake of these guidelines and of sustainable worm control programs in general has been slow and is patchy (43, 50–54). Accordingly, the need for understanding farmer's behavior in parasite control, and more specifically the uptake of these applicable advices, is growing. The understanding of farmers' intention to adopt such sustainable control practices is necessary to create effective strategies for promoting sustainable worm control. A number of behavioral factors influencing farmers' adoption must be considered if recommendations are to be developed and wide acceptance is to be achieved (55–57).

**Table 3:** COWS guidelines (more details see: www.cattleparasites.org.uk).

**Guideline**	**Comment by Taylor (42)**
Work out a control strategy with your veterinarian or advisor.	Specialist consultation as part of herd health planning is an increasing requirement on farms. Worm control programmes for cattle will require on-going consultations.
Use effective quarantine strategies to prevent the importation of resistant worms in introduced cattle.	Bought in cattle can be a potential route of introducing resistance alleles into a non-closed herd
Test for anthelmintic efficacy on your farm	Whilst resistance is still rare in cattle nematodes, treatment failures do occur. It is important to monitor continued efficacy as under dosing can select for AR
Administer anthelmintic drugs effectively	Administer the right dose in the correct way by following manufacturer's instructions
Use anthelmintic drugs only when necessary	Understand the trade-off between tolerating some level of parasitism and minimizing selection for AR. FEC monitoring has an important role
Select the appropriate anthelmintic for the task	Target treatment according to parasites (and their stages) present, based on time of year
Adopt strategies to preserve susceptible worms on the farm	Aim to reduce selection for AR when treating adult cattle, immune older animals or when dosing on low contamination pastures
Reduce dependence on anthelmintic drugs	Alternative control measures include grazing management using sheep or older immune animals

*Table adopted from Taylor (42) p. 67*.

## Farmers' adoption of sustainable control

### Factors influencing the adoption of sustainable worm control practices

The adoption of sustainable strategies is affected by many personal factors, which can be divided into benefits (i.e., beliefs positively affecting behavior) or barriers (i.e., beliefs negatively affecting behavior). The first studies conducted in veterinary parasitology focused mainly on reporting current helminthic control strategies on sheep farms and the technical barriers to the uptake of alternative and sustainable methods. This was a response to limited adoption of the new, sustainable control strategies. Morgan et al. (54) presented a survey of 600 sheep farmers to characterize current practices, and to identify factors correlated with perceived anthelmintic failure. Although most farmers considered helminths to be a problem on their farms, only half of them were concerned about AR and even fewer believed this compromised their current GIN control (54). Furthermore, anthelmintic use was influenced by past experience and perceived reliability of the drugs, along with convenience of use and price (54). Besides, only a minority of the respondents were aware of the local program of sustainable worm control (SCOPS) (54). Low awareness of both the risk of AR and concomitant information campaigns, and positive attitude toward their current use of anthelmintics were accordingly identified as barriers for the adoption of sustainable practices. However, later studies indicate a disconnection between the awareness of AR and on farm problems to nematode control (53). Treatment failure was not seen as a consequence of farmers' own behavior. Consequently, farmers failed to see that AR is challenging their current control practices. Similar experiences were reported for other countries, such as Australia and New Zealand, where AR prevailed much earlier and is now present on most sheep farms (43, 55). These reports concluded on additional barriers, which in turn were more adoption specific, such as complexity of the new GIN control approaches and their compatibility with the current approach, time requirements, and the ability to trial the proposed management practices (43, 58). Moreover, the awareness of such new control approaches was associated with concerns on AR, previous experience with diagnostics and the consultation of professional advisers regarding worm control (59).

Although most literature focuses on small ruminants, some reports have been made for cattle and horses. These indicate a failure in learning the lessons from resistance development in small ruminants (60). Both the cattle and equine industries remained until recently oblivious to the issue of AR, which could explain the reluctant position for changing current practices (52). A study on UK horse owners did establish some concerns on AR, however only a small number were willing to reconsider the use of anthelmintics in their horses (61). McArthur and Reinemeyer (52) allocate some responsibility to cattle practitioners in the US in particular, as they may not have the knowledge to implement evidence-based recommendations toward producers. Though, recent studies investigating UK anthelmintic prescribers indicated a good knowledge of basic helminthology and best practice guidelines for livestock veterinarians (62). Kenyon et al. (37) on the other hand consider the advice given to livestock owners in particular more problematic. These advices can be contradictory, and tend to change depending on scientific knowledge. For example, certain stakeholders still hold on to old control practices, such as fixed treatment schedules, though evidence-based control is now best promoted to livestock owners. However, the latter can only be performed with some knowledge of diagnostic methods and parasitic markers.

These reports provide a description on the factors affecting the uptake of advices and strategies on sustainable GIN control throughout different geographies and animal species. However, the outcome is a tangle of different factors and explanations for farmers' GIN control approach. Indeed, many of these reports are based on opinions and personal experiences with livestock owners, or are simply based on “yes-or-no” questions with immediate relation to farmers' current or future control. This limited behavioral research may result in unsubstantiated hypotheses. Nevertheless, these insights provided important contributions for herding the scientific world toward a paradigm shift regarding farmers' decision making in GIN control. The need for more structured and scientific behavioral research is growing. Therefore, a shift is emerging toward social veterinary epidemiology, a fairly young discipline with contributions from different fields, such as behavioral psychology and sociology (63).

### Lessons learned from social veterinary epidemiology

From a historical perspective, policy makers, researchers, and veterinarians assumed that farmers' decisions were solely based on rational, technical and economic considerations (64). Livestock farming is a business, thus external factors, such as market price and customer demands, as well as costs and returns, influence the decision-making process (65, 66). These rational choices play an important role, but are certainly not the only decisive factors. Correspondingly, livestock farming is intertwined with lifestyle and is often associated with family, hence much of the decisions can be explained through more personal traits of the farmers' social environment (67–69). Poor on-farm adoption of recommendations to decrease disease transmission or enhance biosecurity practices, and low participation in voluntary disease prevention, urged for a better understanding of farmers behavior (70). Personal traits often explain more variation in farm performance than farmers' measurable management practices (71). Therefore, the main goal of social veterinary epidemiology is identifying these traits in order to explain and predict farmer specific behaviors, which mainly consist of socio-psychological factors (e.g., attitude, subjective norms, risk perception) derived from human behavioral and health psychology.

**Figure 1:**
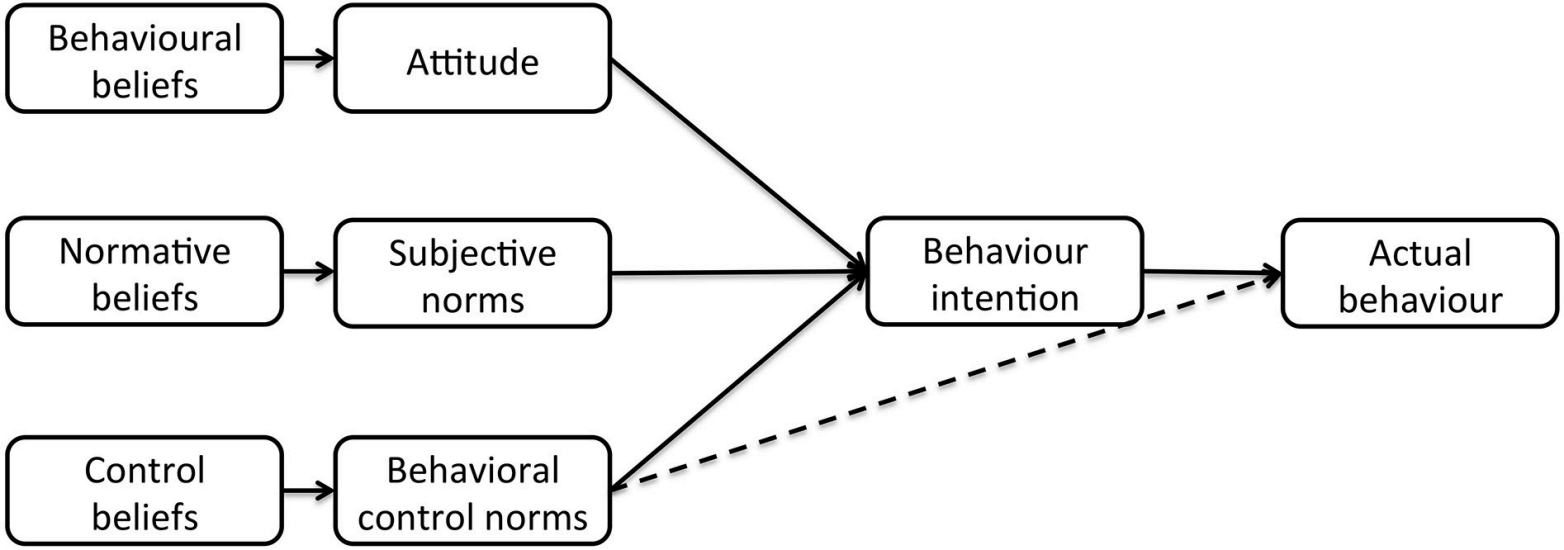
The theory of planned behavior.

**Figure 2:**
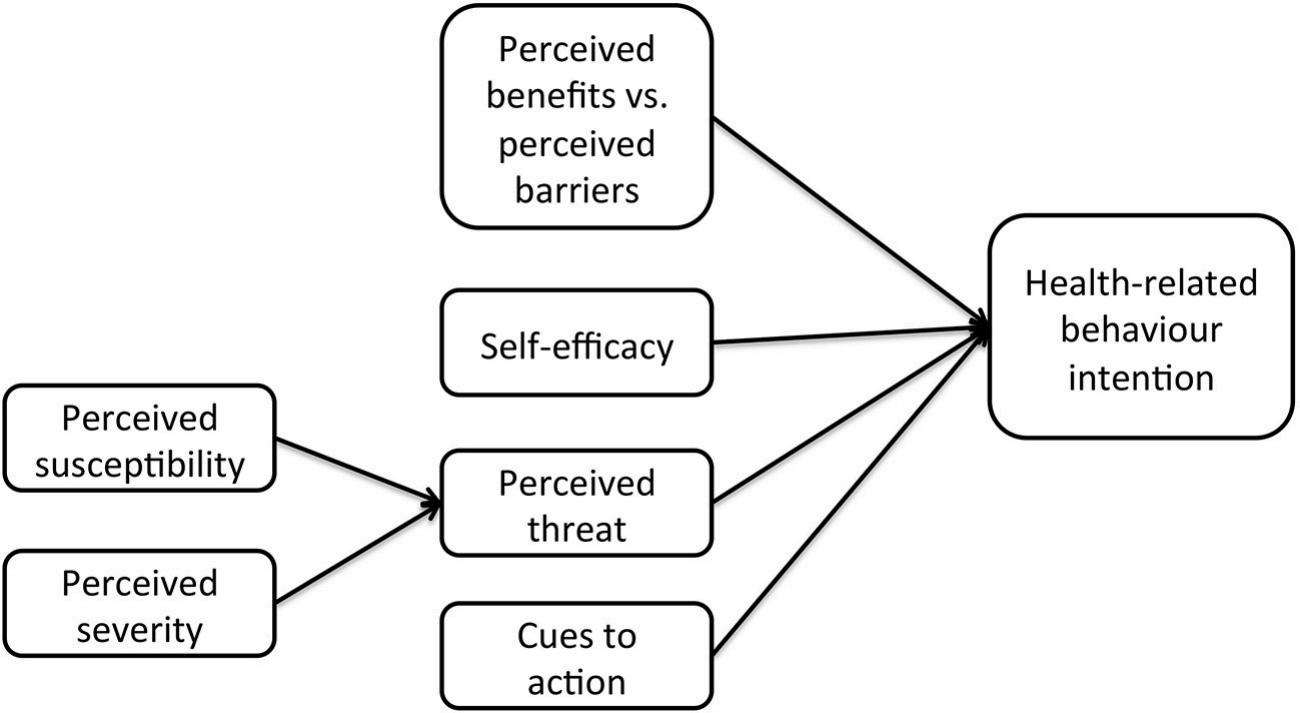
The health belief model.

The incorporation of socio-psychological theories and methodologies with traditional epidemiologic approaches has been proven useful for exploring cattle farmers' intentions and behaviors. The two most commonly used theories are the *Theory of Planned Behavior* (TPB, Figure 1) (72) and the *Health Belief Model* (HBM, Figure 2) (73).

These theories suggest a bridge between socio-psychological factors, which are formed by a person's beliefs, and behavior. Within the TPB, behavior is determined by behavioral intention, which is subsequently determined by attitude (i.e., positive or negative evaluation of the particular behavior based on the expected outcomes), subjective norms (i.e., perception of the expectation of significant others in performing that behavior) and perceived behavioral control (perceived ability to perform a specific behavior).

The HBM suggests that people's beliefs about health problems and related treatment programs describe the engagement in health-promoting behavior (74). The mechanisms behind the HBM are similar to those of the TPB, with the addition of health-specific factors, such as perceived susceptibility (i.e., perception of the vulnerability to danger or harm), perceived severity (i.e., perception of the impact of the risk and its harm), and cues-to-action (triggers for prompting engagement in health-promoting behaviors). The implementation of such models gives a more structured view and justified prediction of farmers' behaviors.

These, or similar, approaches have been used to examine a wide range of animal health-related behaviors, such as the control of mastitis (69, 71, 75), Johne's disease (76–78), foot-and-mouth disease (79, 80), lameness (81, 82); the implementation of on-farm biosecurity (83, 84); vaccination strategies (85–87) and antimicrobial usage (88–90). However, due to the specificity of behaviors and the context of the farmers according to different diseases and geographies, it is impossible to provide a “one-size-fits-all” model and explanation. It is therefore necessary to study GIN control in particular, and more specifically the adoption of sustainable worm control.

### Social veterinary epidemiology for GIN control

The TPB and the HBM were merged in a novel framework to predict dairy farmers' adoption intentions of diagnostic methods for GIN control in Flanders (Belgium) (91). Farmers' positive attitude for diagnostics and the perceived pressure of the subjective norms were the main drivers of this intention. Furthermore, farmers' positive attitude toward anthelmintic drugs and concomitant preventive use, was identified as a barrier for possible uptake. AR on the other hand was not perceived as a risk, and had no effect whatsoever on the adoption intentions of the dairy farmers. A study of Rose Vineer et al. (92) in UK horse owners obtained similar results, but identified perceived knowledge as an additional driver for adoption intention of diagnostic methods. Moreover, perceived knowledge increased the intention to use diagnostics via attitudes, subjective norms, and perceived control. Knowledge was again identified as an important factor for the uptake of sustainable control practices (i.e., SCOPS) by Scottish sheep farmers (57). In contrast to the previous studies, Jack et al. (57) found AR risk perception to have an effect on the SCOPS practice uptake, and the confirmation of AR by a diagnostic test/external advisor had the largest effect on this uptake.

These studies connected socio-psychological factors to the adoption intention of sustainable control and uptake of advises, which was lacking from previously mentioned literature. To do so, quantitative data were obtained through surveys and analyzed with modeling techniques, such as structural equation modeling. Although this gave an overall understanding and possibilities for adoption, the interpretation remained fairly superficial, because important factors (e.g., subjective norms) were identified, but specific factors (e.g., the veterinarian vs. peer farmers, vs. family) underlying these drivers were not further explored. Accordingly, in-depth analyses are necessary to determine farmers' beliefs and motivations underlying these socio-cognitive factors. Qualitative research is more suited to understand these factors, as it explores values and perspectives that are more difficult to grasp by using quantitative self-reports.

In addition, socio-cognitive frameworks based on the TPB or HBM predict behavior intentions rather than actual behavior. The “intention-behavior gap” is an important concept in the domain of behavioral and health psychology (93). Numerous different theories in this domain explain why behavioral intentions do not automatically lead to consequent actual behavior (94). Concerning farmers' behavior, several factors were suggested to form a bridge between intention and behavior, such as habits, the impact of the community and culture (65, 68, 95). Nevertheless, this intention-behavior gap has rather been neglected in the field of veterinary parasitology. One study took these limitations into account (96) and presented a model for dairy farmers' adoption of sustainable GIN control methods. The model consisted of three different phases: adoption intention, actual adoption and maintenance, and served as an extension of the previously tested framework (91). Data were collected through semi-structured interviews with dairy farmers. Low infection awareness and low priority (“top of mind”) of the disease were identified as important barriers for farmers' positive intentions toward sustainable GIN control. Secondly, different types of motivations influence different sorts of behavior: i.e., sustainable behavior, such as use of diagnostics is influenced by moral motives, while management behavior, such as anthelmintic treatment is raised by more economic motives. Thirdly, farmers' behavior is guided by two important social norms: the opinion of their veterinarian and their fellow farmers. However, farmers hold an incongruent relationship with both norms throughout the different stages of behavior: they do not value other farmers' opinions as a positive reference (intention phase), but they do follow and mimic their behavior as a group (action phase). The veterinarian was identified as the most important positive reference, but also the responsible actor for disease control, and GIN control in particular. As such, the farmers did not hold themselves responsible for implementing sustainable control strategies. Finally, not only performing, but also maintaining behavior was important to fully address the adoption of sustainable worm control. To perform and maintain the adoption on farm, planning was suggested as an important contribution, which could help to surmount other suggested barriers for actual adoption, i.e., habits and responsibility (96).

## Communication as a first step toward change

The end-goal of most of the above-mentioned literature in social veterinary epidemiology is to exploit knowledge on farmer's current (and future) behavior in targeted communication campaigns and seed for a motivational change in behavior. However, the usability for translating results from sociology-type studies into communication strategies is barely explored for GIN control in specific, and for animal health in general. Some of the previous work makes grounded suggestions for communication strategies, but their effectiveness remains unconfirmed. Woodgate and Love (43) propose to enhance the visibility of the problem and concomitant positive outcome when implementing best practice management on sheep farms in Australia (Wormboss). Moreover, evidence of potential economic loss should provide a powerful message regarding the need for effective control programs (55). McArthur and Reinemeyer (52) suggest that farmers will only be willing to abandon their historical practices if they can be convinced through economic analyses and scientific evidence. The extension campaigns should also focus on the relative advantage, complexity and compatibility of the sustainable methods, and the ability to trial the proposed change (43). This can be provided through targeted education and practical demonstrations (52).

However, as stated above, the effectiveness of the above suggestions has not yet been demonstrated. One possible way to do so is through trialing public service announcements (PSA) to a test audience according to experimental studies. A PSA is an advertisement in the public interest with the objective of raising awareness, and eventually changing public attitudes and behavior toward a social issue. Vande Velde et al. (97) created and validated PSAs to change dairy farmers' behavior intentions based on the knowledge of previous social epidemiological studies. The study was set up to create awareness of AR, without evoking too many negative responses from the farmers. A humorous PSA with two-sided argumentation appeared to be effective, as it decreased negative responses (97). However, the more simplistic message without humor and only one-sided argumentation was also found effective, illustrating the importance of communication research before sending out a message to the farmers. Awareness campaigns using short messages (e.g., PSA) can now benefit from this scientific evidence. However, the study was a single case experiment and the result should be considered more a trend. Additional case studies could confirm the effectiveness of the different PSAs on changing farmers' intentions. Moreover, the measurement was limited to the intention (i.e., motivational) level of the farmer and future studies should contribute some support for actual change and maintenance of the behavior. Finally, such messages are set up to create awareness, which only implicates a small knowledge transfer. PSAs should trigger the farmer to take action and gather information applicable for the own farm.

## Discussion

There is a substantial gap in the literature on cattle farmers' behavior, and livestock owners in general, when it comes to GIN control. Although the risk of AR is well-established in small ruminants, and emerging for cattle and horses, the uptake of advises for sustainable control remains limited and little actions have been taken from the scientific world to understand the underlying mechanisms. Research was primarily focused in finding best management practices to overcome this emerging risk. While further evidence is required on the impact of best management practices on various sustainability criteria, it is time to promote and translate current insights into applicable advises. Up until now, much of the literature dedicated to understand the uptake of animal health advises, was based on descriptive assessments and not grounded in sociologic research methods. Few studies implemented social veterinary epidemiology to gain a better insight into the livestock owners' mind (57, 91, 92, 96), however, some limitations should be addressed.

### Limitations of social veterinary epidemiology

**Figure 3:**
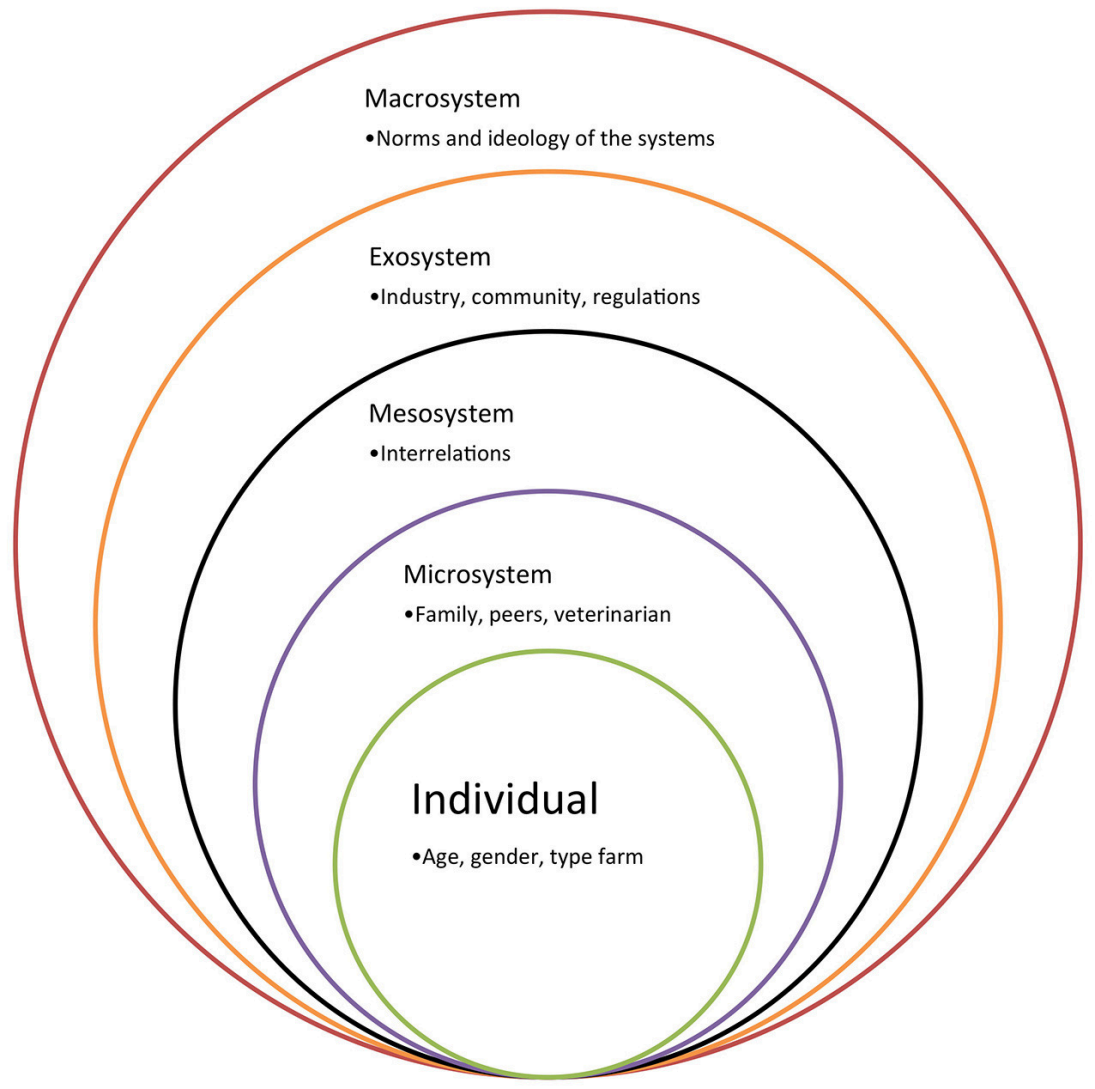
The ecological systems theory.

The results obtained through such socio-psychological behavioral models take more intrinsic and individual drivers into account, without explicit inclusion of external factors or economic assessments. Besides these socio-psychological determinants, there are often other factors influencing farmers' decisions that are not (or less) internally driven (98). Therefore, farmers' behavior should not be considered guided only by the ego, but also by other, extrinsic circumstances, which (s)he has less control over (65, 95). In Brofenbrenners' Ecological Systems Theory (Figure 3; 1977) the individual is placed at the smallest level of a greater system. Five environmental levels interact with one and other, and eventually lead toward integrated decisions of the individual. Correspondingly, and for agricultural purposes in particular, the Agricultural Innovation Systems (AIS) thinking has become an increasingly applied method to analyze and comprehend technological, economical and institutional change (99). Farmers' decisions are considered the result of a process of networking and interactive learning among different environmental levels and actors [e.g., other farmers, veterinarian, traders, government, animal health organizations; (100)].

On the other hand, livestock farming is a business, thus external economic factors also influence the decision-making process. Results from behavioral research with private horse owners (92) were very similar to those of dairy farmers, although both populations are moved by different context. Hence, the results obtained through these socio-psychological models take more intrinsic and individual drivers into account, without inclusion of economic rationality. These behavioral models, for farmers in particular, could benefit from the inclusion of production economics. In particular, economic models are established to optimize GIN control approaches from an economic perspective (101). Incorporating all the above could provide novel insights in farmers' behavior and present a new view of decision making where socio-psychological factors and economic factors are balanced, together with the regulatory obligations in order to achieve improved animal health management. Subsequently, more targeted and farm-specific advices could be established with an eye on sustainable and profitable results.

**Figure 4:**
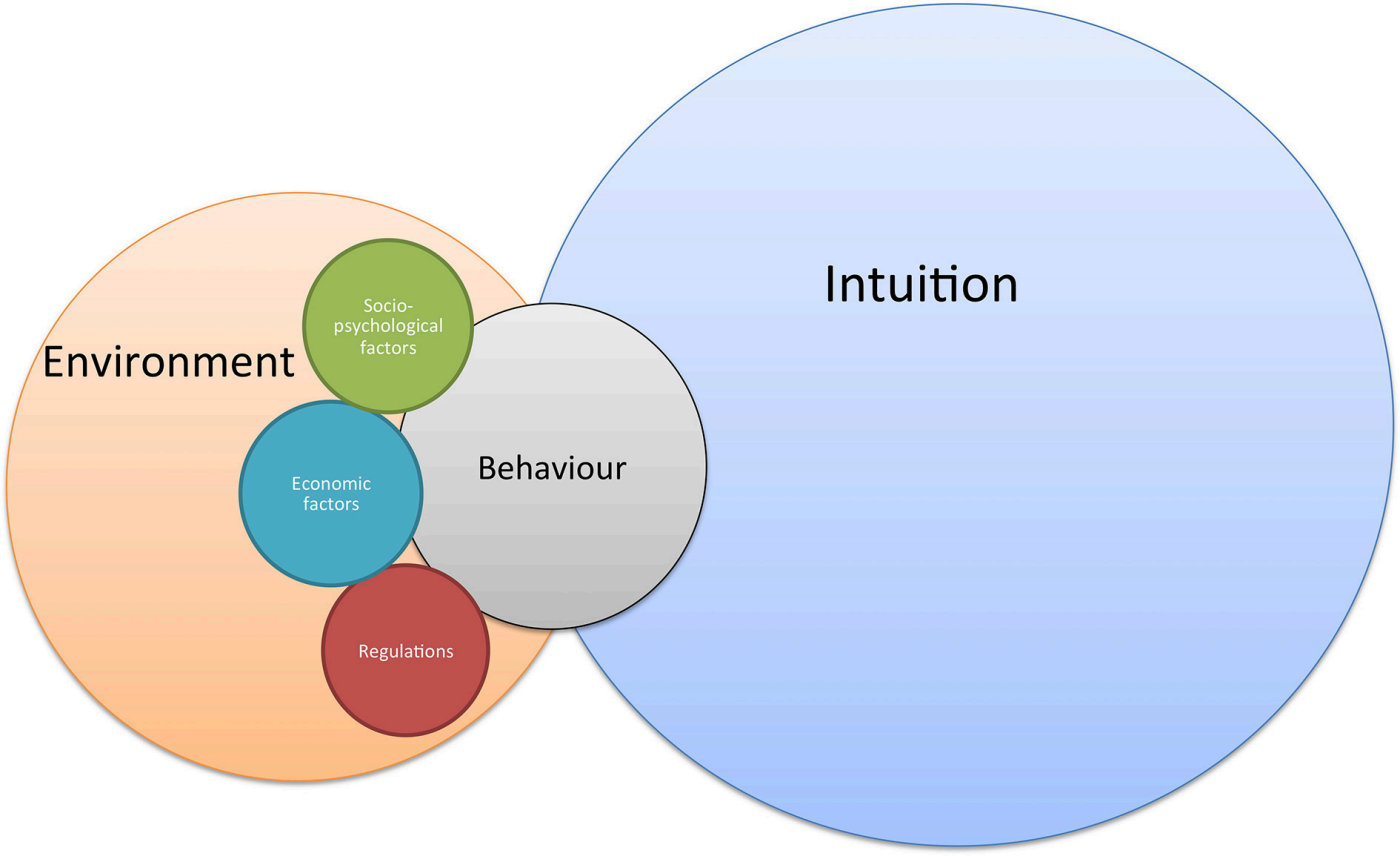
General framework on farmers' behavior, driven by intuition (unconscious) and conscious decisions.

Finally, farmers' behavior is just regular human behavior, and the majority of the decisions are based on intuition and unconscious paths (102). The framework presented in Figure 4 takes into account that research into farmers' decision-making is not solely based on one or another discipline but consists of a tangle of different philosophies and research areas. Conscious decisions depend on the farmers' environment (e.g., community, industry, institutions), which place the individual farmer into the smallest level of a larger perspective (103). On its turn, conscious decision-making can be divided in three types of behavior (104), initiated by three different motivations: compulsory behavior based on regulation [external motivations; (105)] incentive-driven behavior based on economic rationality (both external and internal motivations), and voluntary behavior driven by socio-psychological factors [internal motivations; (105)].

### Limitations in communication

Communication strategies studied for changing farmers current GIN control, remained limited to PSA's with short persuasive advertisements. Moreover, the experiment measured farmers' intentions, which only partially explain adoption. Changing a whole population's behavior is not simply established through short persuasive messages, although it is generally considered as a good start (106). Farmers would also benefit from more personal communication and educational programs in order to actually change their current, unsustainable behavior, and to establish maintenance and behavior change in the long run. However, this top-down approach is still an archaic look on knowledge transfer and shift should focus toward other paradigms. Wilson et al. (107) suggested a systems approach where knowledge is built and shared through equal involvement of different stakeholders. In agricultural extension (i.e., application of scientific knowledge in agricultural practices through farmer education), different paradigms are proposed with an eye on different methods: paternalistic (i.e., top-down information exchange) vs. participatory (i.e., bottom-up) and outcomes: persuasive (behavior change) vs. educational (building knowledge). Empowered participatory governance is a method to create regulation in a bottom-up approach by the population itself (108). It is an exercise among several different actors (stakeholders) of a population, which contribute ideas and solutions in a democratic, problem-solving debate (109). The outcome of this debate is self-sustained regulation, which becomes a norm when fully accepted by the population. Policy and governance regulation is still the most effective way to change a populations' behavior (e.g., taxes on alcohol and cigarettes, speed limitation). However, governance regulation is not always applied, or foreseen in the near future. Therefore, empowered participatory governance could be a good alternative for guiding behavior that is not immediately regulated by the government. Because this lack of policy in anthelmintic prescription and usage, farmers could benefit from self-sustained regulation. Therefore, empowered participatory governance in particular, or participatory agricultural extension in general, seems promising for tailoring future strategies for sustainable GIN control.

Considering behavior is not always consciously driven (see Figure 4), other methods, focusing on unconscious paths (or heuristics) can also be promising tools for future campaigns. For example, nudging is a choice of architecture that alters people's behavior in a way without forbidding any options or significantly changing their economic incentives. It can be considered as a small push to guide people in the rightful behavior (110). Although this is a promising approach, the long-term effects of nudging have not been proven yet. Due to its unconscious character many presume that its positive effect will vanish along with the architectural primer if removed (111). Therefore, the method of choice for changing farmers' behaviors remains a more cognitive persuasion. Nevertheless, a mixture of both unconscious (e.g., nudging) and conscious (e.g., processing and generating information) may eventually have the best effect on changing farmers' behavior in the long run (109).

## Conclusion

Sustainable approaches for GIN control have been researched and introduced to cope with the emerging AR. This was a major focus of research in parasitology in the last decades. These tools were provided to the livestock community in developed countries, however adoption remained slow. New research in this domain is set up to understand why this adoption is limited. Until recently, literature on farmers' uptake of GIN control was scarce and only a few papers have introduced socio-psychological factors to explain or predict farmers' behavior. Therefore, more socio-psychological studies on farmers' GIN control are needed to obtain a general, but profound view of this behavior. Besides, we should look further than the rational and conscious decisions of farmers and include other theories and methods to identify additional drivers of farmer's behavior, such as intuition. Moreover, it is important to train veterinary parasitologists into the field of social sciences, to increase their participation in social veterinary epidemiology. Finally, the results of this research should be translated into practical advice and disseminated to veterinary extension services and the end-users, i.e., farmers and veterinarians.

## Author contributions

FV and EC contributed to the conception and design of the review. FV wrote the first draft of the manuscript. FV, EC and JC contributed to manuscript revision and read and approved the submitted version.

### Conflict of interest statement

The authors declare that the research was conducted in the absence of any commercial or financial relationships that could be construed as a potential conflict of interest.
